# Publication trends of research on diabetes mellitus and T cells (1997–2016): A 20-year bibliometric study

**DOI:** 10.1371/journal.pone.0184869

**Published:** 2017-09-19

**Authors:** Ye Gao, Yiran Wang, Xiao Zhai, Yifei He, Rong Chen, Jingjing Zhou, Ming Li, Qijin Wang

**Affiliations:** 1 Graduate Management Unit, Changhai Hospital Affiliated to theSecond Military Medical University, Shanghai, China; 2 Department of Oncology, Changhai Hospital Affiliated to the SecondMilitary Medical University, Shanghai, China; 3 Department of Orthopedics, Changhai Hospital Affiliated to the SecondMilitary Medical University, Shanghai, China; 4 Department of Endocrinology, Changhai Hospital Affiliated to the SecondMilitary Medical University, Shanghai, China; Baylor College of Medicine, UNITED STATES

## Abstract

**Introduction:**

Diabetes Mellitus (DM) is a huge burden for human health. Recent studies show the close relationship between DM and T cells. We investigated the trend in DM and T cells research.

**Methods:**

Using the Web of Science database, we searched the publications on DM and T cells in 1997–2016, and studied the source data using bibliometric methodology. Excel 2016, GraphPad Prism 5, and VOSviewer software were used to analyze the publication trend in DM and T cells research.

**Results:**

We found a total of 1077 publications with 38109 citations up to January 23, 2017. The highest contribution came from the United States, with 48.38% of the publications, 61.44% of the citations and the highest H-index (74). China had the 5th place for total publications, but ranked 11th both for citation frequency (604) and H-index (13). The inflection point of the global DM and T cells publications was in 2000. Journal of Immunology published the most related articles (164). Santamaria P. was the leading scholar in this field with the most publications (35). The keywords “regulatory T cell” and “autoimmune diabetes” were mentioned more than 300 times. Furthermore, type 2 (T2)DM, T cell immunoglobulin and mucin domain (TIM) and obesity are becoming popular research topics in this field.

**Conclusion:**

The quantity of publications on DM and T cells grew rapidly around year 2000, but has relatively decreased recently. The United States had the leading position in global research. There was a discrepancy between productivity and quality of publications from China. Latest progress is most likely first published by the Journal of Immunology. Santamaria P., Roep B.O. and Peakman M. were the pioneer scholars in this field. Most researchers have focused on “regulatory T cell” and “autoimmune diabetes” research. In future, T2DM, TIM and obesity may be the popular areas.

## Introduction

Diabetes mellitus (DM) is a glucose metabolism disease characterized by chronic hyperglycemia resulting from defects in insulin secretion, insulin action, or both[1]. Type 1 (T1)DM results from an absolute deficiency in insulin caused by the failure of secretion by the pancreas, while type 2 (T2)DM is characterized by insulin resistance and relative insulin deficiency, either or both of which may be present at the time diabetes is diagnosed. DM poses a great threat to human health as well as a huge socioeconomic burden for governments. According to the updated data from the international diabetes federation (IDF), the estimated global prevalence of DM reached 8.8% in 2015 and 12% of global health expenditure was due to DM in that same year[2]. Attempts to understand the pathogenesis of DM are ongoing. T1DM was traditionally considered an autoimmune disease. Early research demonstrated that T cells were involved in various pathogenic steps in T1DM, including the initiation of insulitis and the injury to β cells[3]. Moreover, recent research has also discovered the role of T cells in the development of insulin resistance[4] and various complications in T2DM, including atherosclerosis[5], nephropathy[6] and neuropathy[7]. Thus, T cells may be a key component in the pathogenesis of DM as well as a potential diagnostic and therapeutic target. However, studies on quantitative and qualitative characteristics of global research on DM and T cells are limited and this topic needs more attention.

Bibliometric analysis provides information about the trend in research activity over time and compares the contributions of scholars, journals, institutes and countries[8]. It uses the literature system and literature metrology characteristics as research objects and analyzes the publications quantitatively and qualitatively[9]. It has been increasingly popular because of its application in policy and clinical guidelines making and has been successfully used in evaluating the research trend in cardiovascular disease[10], respiratory medicine[11], gastrointestinal diseases[12] and DM[13].

Our research attempted to provide all-round insights on the current state of global DM and T cells research. The distribution of the publications was analyzed as well as keywords and references to better understand the global trend of research and to discover the popular topics in this field.

## Materials and methods

### Data sources and selection criteria

**Fig 1:**
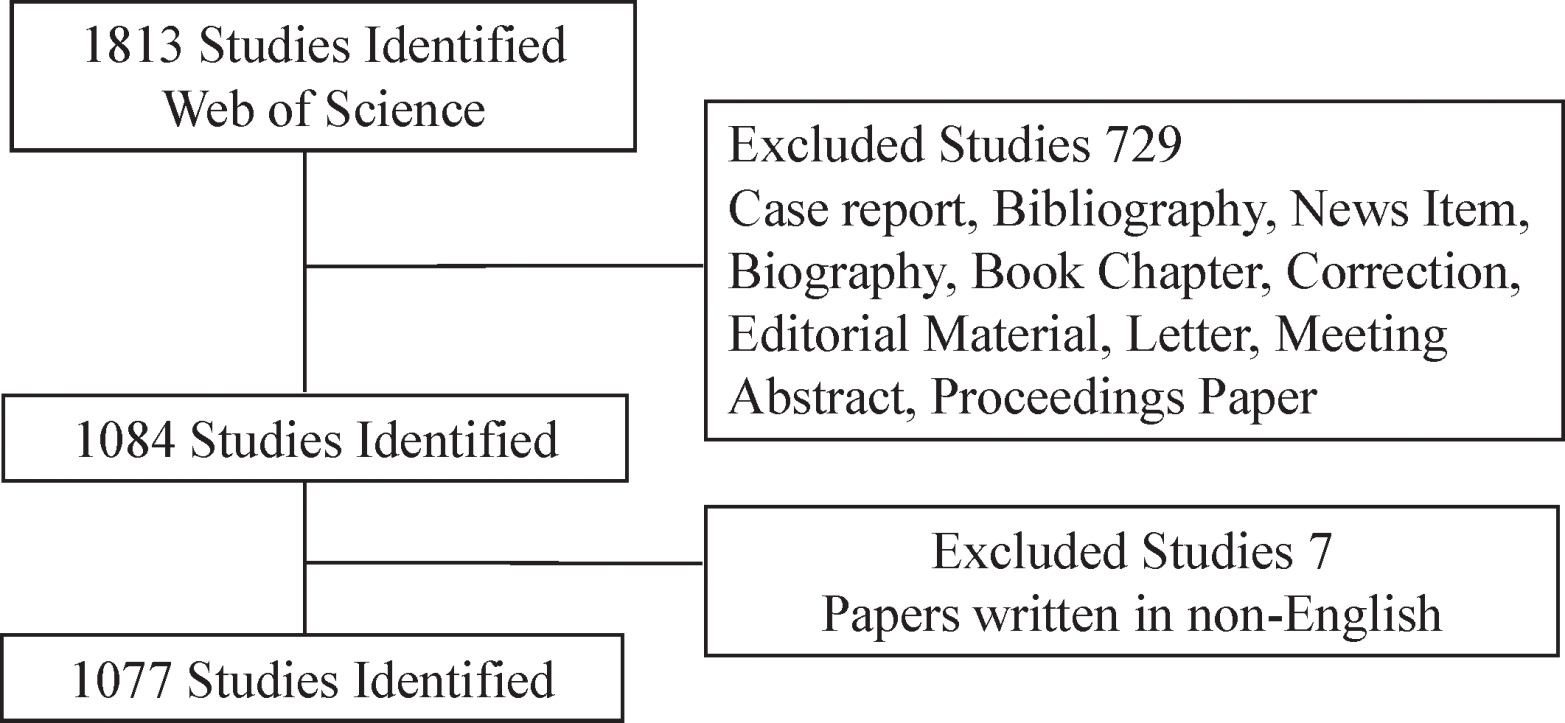
Demonstration of the retrieval process. The process of retrieval and exclusion criteria.

The Science Citation Index-Expanded (SCI-E) of Thomson Reuters’ Web of Science provides prepared and comprehensive data of publications and is considered the optimal database for bibliometric analysis[14]. The literature search was performed in the Web of Science database. The manuscript types were restricted to original articles and reviews. All searches were performed on a single day, January 23, 2017, to avoid changes in the number of publications and citations as much as possible. The retrieval strategy was: TI = (T cell* OR T lymphocyte*) AND TI = diabetes* AND Language = (English). Articles and reviews that were normally peer-reviewed were included, but all others were excluded. The total retrieval process was demonstrated in detail in Fig 1.

### Data collection

The data of all eligible publications including title, year of publication, authors' names, nationalities, affiliations, name of publishing journal, keywords, abstract, times of citation per year and H-index per country were downloaded from the internet and extracted carefully and independently by two authors (Ye Gao and Yiran Wang). The data were imported into Microsoft Excel 2016, GraphPad Prism 5 and VOSviewer and analyzed both quantitatively and qualitatively.

### Bibliometric analysis

The intrinsic function of Web of Science was used to describe the aforementioned basic characteristics of eligible publications. The H-index was designed as a measure of scientific research impact. The index of H indicates that a scholar or country has published H papers and each of which has been cited in other publications at least H times. Therefore, the H-index reflects both the number of publications and the number of citations per publication. Relative research interest (RRI) was defined as the number of publications in a certain field divided by all-field publications per year. Impact factor (IF) of all journals were obtained from the Journal Citation Reports of 2016. GraphPad Prism 5 was used to draw the time curve of publications using the logistic regression model: *f(x)* = *c*/[*1*+*a*×*exp*(-*b*×(*x-1996)*)]. In this formula, the independent variable *x* refers to the year and the *f(x)* refers to the cumulative amount of publications. The formula *T* = *lna/b+1996* was used to calculate the inflection point defined as the time the growth rate of publications changes from positive to negative. Furthermore, we used VOSviewer to analyze the relations between highly cited references and productive authors as well as to create the knowledge maps of cited references and keywords related to DM and T cell research.

## Results

### Contributions of countries and regions to global publications

**Fig 2:**
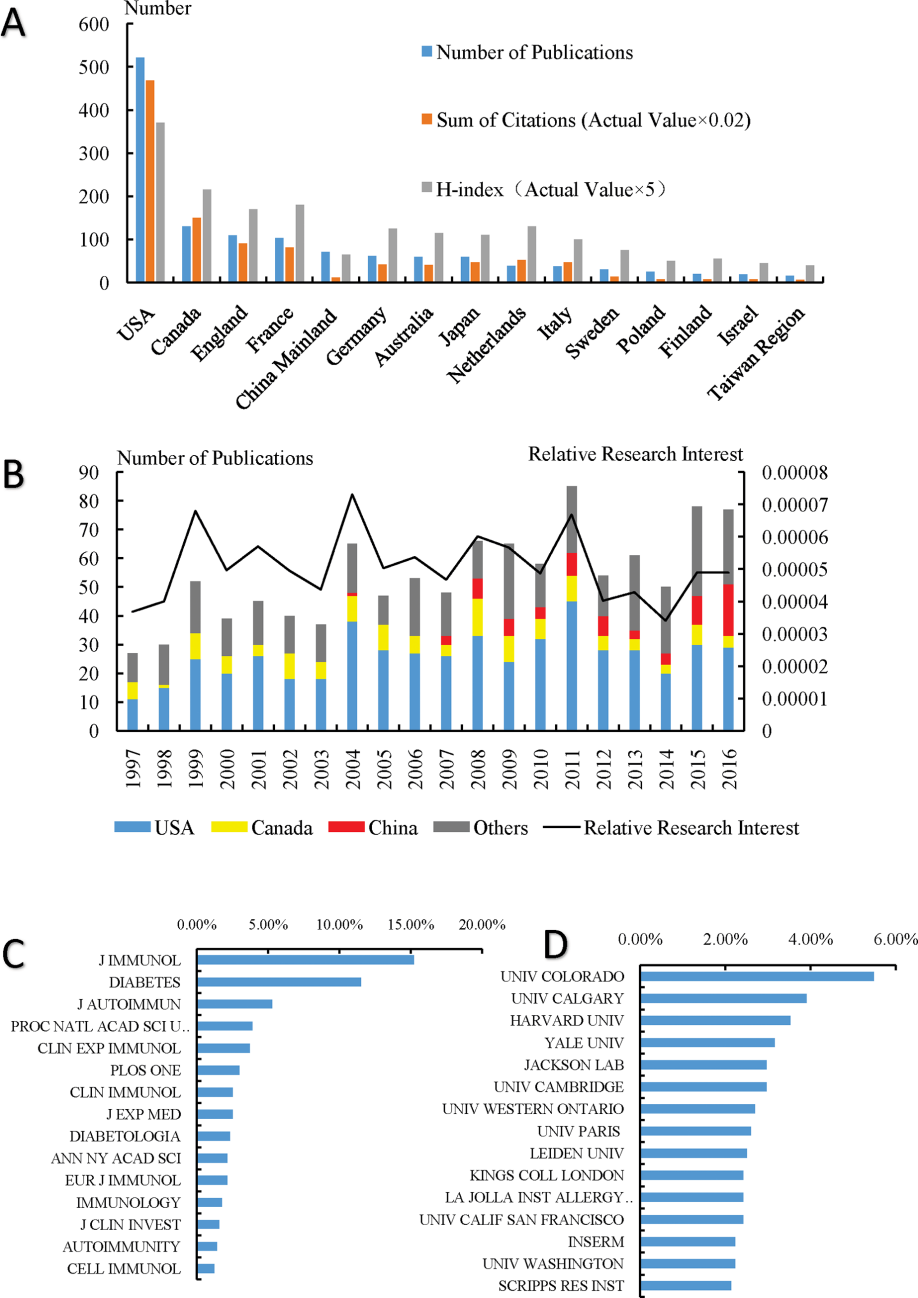
Contributions of different countries, regions, journals, institutes to DM and T cells research. (A)The number of publication, citations and H-indexes on DM and T cells research of the top 15 countries and regions; (B) Relative research interests and single-year publication numbers on DM and T cells research; (C) Distribution of top 15 journals that published on DM and T cells research; (D) Distribution of top 15 institutes focusing on DM and T cells research.

Both the quantity and quality of publications were measured to evaluate the contributions of different countries and regions. A total of 1077 articles from 1997 to 2016 met the search criteria. The United States had the first place when ranking for the quantity of publications (521, 48.38%), followed by Canada (130, 12.07%) and the United Kingdom (109, 10.12%). China had the 5th place with 71 publications accounting for 6.59% of all publications. The overall number of citations of publications was 38109 since 1997 (32772 times without self-citations). The cited frequency per publication was 35.38 times. The United States had the most citations with a number of 23413 (61.44%) and an H-index of 74. Canada ranked 2nd with the citation number of 7484 and an H-index of 43. Though the number of publications of China ranked 5th, the number of citations and the H-index both ranked 11th (Fig 2A). Most research was published in 2011 (85, 7.89%) when examining the amount of publications per year. In the last 10 years, a significant trend of global publications per year has not been found and the RRIs of this field have been fluctuating around 0.005% with a range from 0.0034% to 0.0067%. The proportion of Chinese publications in global research has been increasing (Fig 2B).

### Journals publishing DM and T cells research

More than half of the publications in this field were published in the top 15 journals (625, 58.03%). The Journal of Immunology (IF = 4.99, 2016) published the most studies with 164 publications. There were 4 articles in Nature (IF = 38.14, 2016), 5 articles in Nature Immunology (IF = 19.381, 2016) and 1 article in Lancet Diabetes & Endocrinology (IF = 16.32, 2016) on DM and T cells research. The top 15 journals that published the most studies are listed in Fig 2C.

### Institutes publishing DM and T cells research

Publications from top 15 institutes accounted for 43.64% of all literature on DM and T cells research. The University of Colorado had the highest number of publications with a total of 59, thereby accounting for 5.48% of all published literature in this field. In the list of the top 15 institutes, 8 are from the United States, 2 are from Canada, 2 are from the United Kingdom, 2 are from France and 1 is from the Netherlands (Fig 2D).

### Number of publications in time

**Fig 3:**
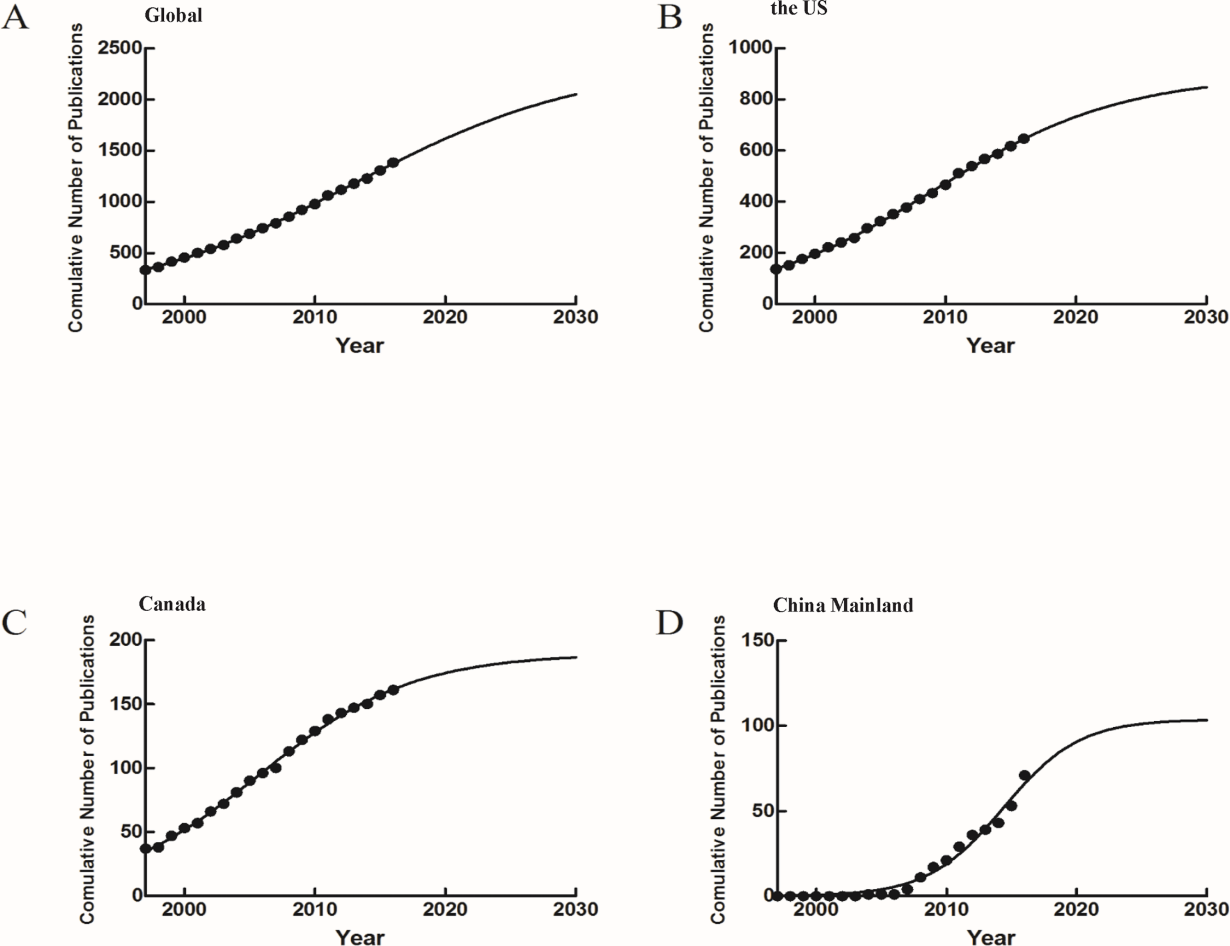
The logistic model fitting curves of growth trends of DM and T cells publications. (A) Global; (B)United States; (C)Canada; (D)China.

The logistic regression model was used to create the time curve of the cumulative number of publications from which the inflection point could be derived and a future trend could be predicted. The inflection point (the time point that the growth rate of the number of publications changed from positive to negative) of global publications was in 2000 as derived from logistic model fitting (Fig 3A). The inflection points for the United States and Canada were both in 1999 (Fig 3B and 3C), while the inflection point for China was in 2008 (Fig 3D). In the future, the cumulative numbers of related literature from the United States, Canada and China will continue to increase, but probably at a much lower rate.

### Authors publishing DM and T cells research

A total of 242 publications were from the top 10 authors, accounting for 22.47% of all publications related to the field. The 3 authors who published the most research were Santamaria P. with 35 publications on DM and T cells, followed by Roep B.O. with 30 publications and Peakman M. with 28 publications (Table 1).

**Table 1:** Top 10 authors with the most publications related to DM and T cells research.

Author	No. of Papers	Country	Affiliation
Santamaria P.	35	Canada	Julia McFarlane Diabetes Research Center, University of Calgary
Roep B.O.	30	Holland	Department of Immunohematology and Blood Transfusion, Leiden University
Peakman M.	28	the UK	Department of Immunobiology, King's College London
Haskins K.	27	the US	Department of Immunology and Microbiology, University of Colorado
Serreze D.V.	26	the US	The Jackson Laboratory, BarHarbor, Maine
Wong F.S.	23	the UK	Division of Infection and Immunity, Cardiff University
Bluestone J.A.	21	the US	Diabetes Center, University of California
Boitard C.	18	France	Inserm UMR 1016, Institute Cochin
Mallone R.	18	France	Inserm U986, DeAR Lab Avenir, Saint Vincent de Paul Hospital
Delovitch T.L.	16	Canada	Department of Microbiology and Immunology, University of Western Ontario

### Analysis of references in DM and T cells publications

**Fig 4:**
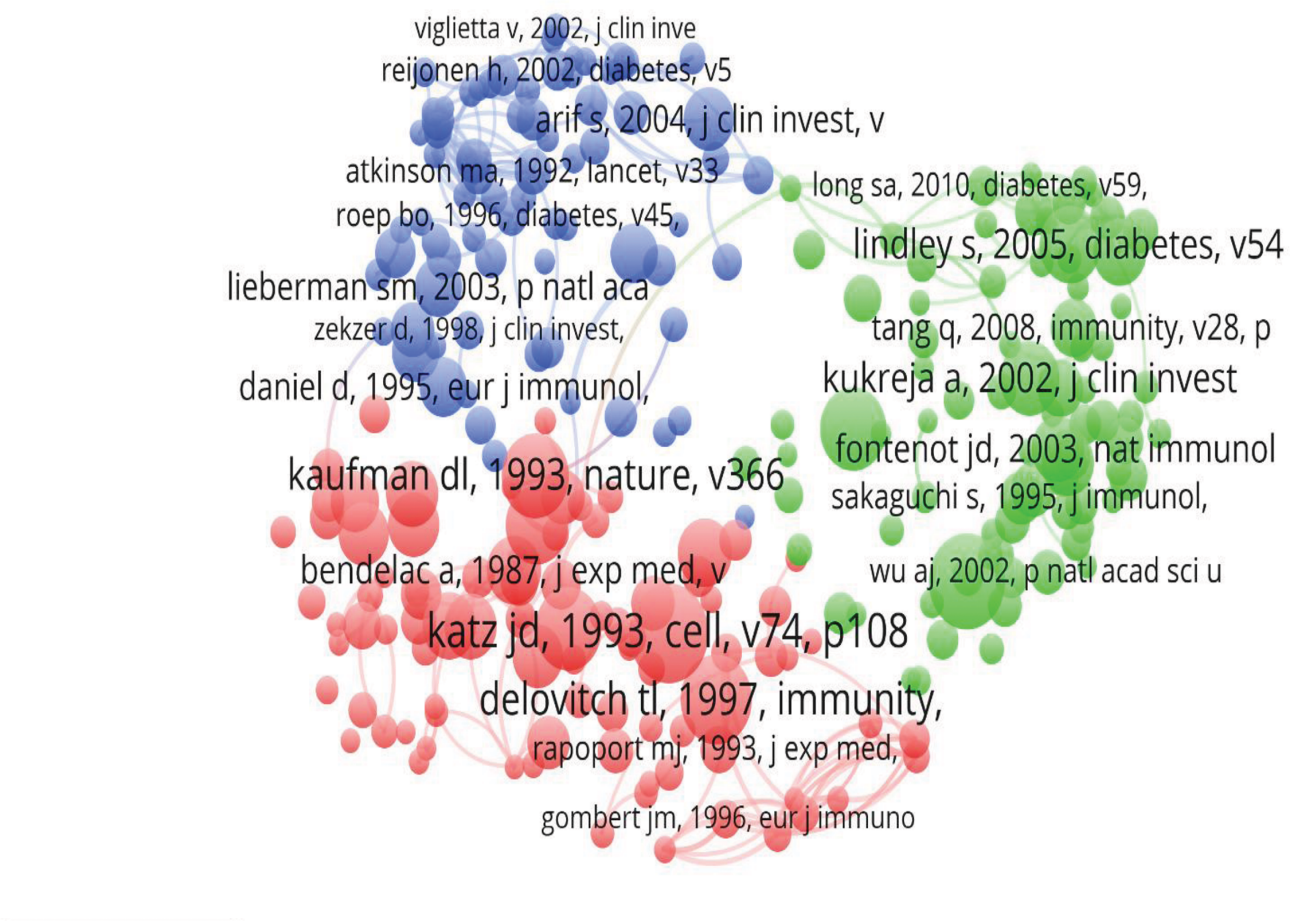
Analysis of the top 245 references. Mapping of the top 245 co-cited references of 1077 publications on DM and T cells research. Due to the large number of cited references, only publications that were cited more than 20 times (N = 245) were included for analysis. (Note: The line between two points in the figure represents that both publications were cited in one publication. The thicker the line, the closer the link between the two publications).

Analysis of references was performed to examine the relations between references and to classify them into different categories. The top 245 references with more than 20 citations were selected for analysis of references by VOSviewer. The references included in the analysis were divided into 3 clusters. The first cluster included 90 publications and mainly focused on the autoimmune process in DM. The second cluster included 82 publications and focused on regulatory T cells in DM. The third cluster contained 73 publications on treatment (Fig 4) (S1 Table).

### Analysis of keywords in DM and T cells publications

**Fig 5:**
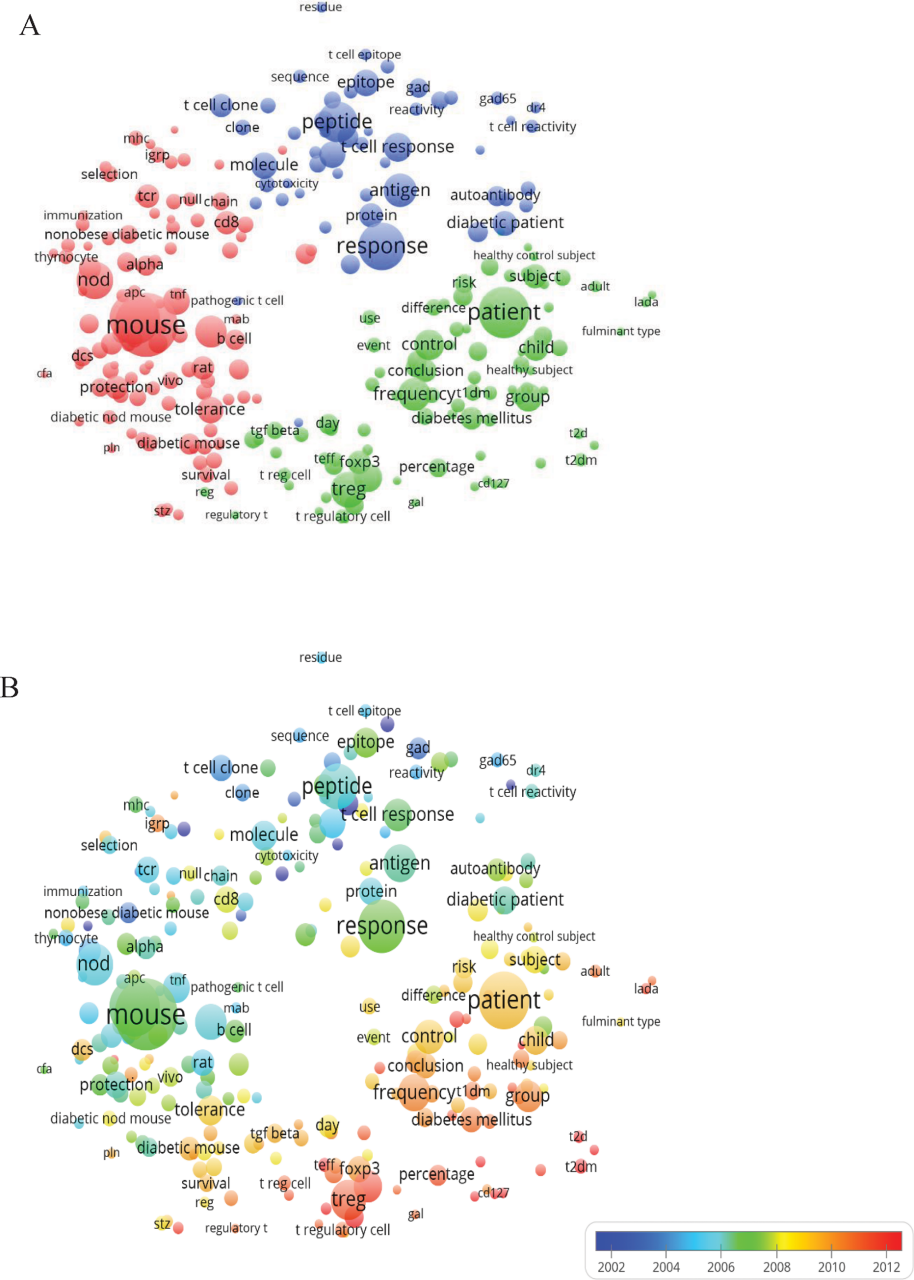
The analysis of keywords. (A) Mapping of the keywords in DM and T cells research. The words were divided into 3 groups according to different colors generated by default; (B) Distribution of keywords according to their time of appearance. The blue color means early appearance and red colored keywords appeared later. Two keywords co-occur if they both occur on the same line in the corpus file. The smaller the distance between two keywords, the larger the number of co-occurrences of the keywords.

The purpose of analysis of keywords is to discover directions and popular topics in research and has proven to be important for monitoring the development of science and programs[15]. Keywords (defined as words that are used more than 20 times in titles and abstracts in all publications) used in the 1077 publications were analyzed using VOSviewer. As shown in Fig 5, the 245 identified keywords appeared at a total of 23136 times and were classified into the 3 clusters: “pathogenesis”, “clinical research” and “treatment” (Fig 5A). In the “pathogenesis” cluster, frequently used keywords were: non-obese diabetic (NOD) mouse (1468 times), autoimmune DM (304 times), insulitis (232 times), tolerance (214 times), and T cell receptor (TCR) (172 times). For the cluster of “clinical research”, the primary keywords were: patient (795 times), regulatory T cell (Treg) (641 times), frequency (328 times), control (276 times), and group (236 times). For the “treatment” cluster, the main keywords were: response (674 times), peptide (503 times), antigen (344 times), T cell response (257 times), and epitope (217 times). The results demonstrated that the most prominent fields of DM and T cells research included 3 directions (S2 Table).

Keywords were color coded by VOSviewer based on the average time they appeared in the 1077 related publications (Fig 5B). The blue color means the keyword appeared early and red colored keywords appeared later. Before 2004, in the early stage of DM and T cell research, the main popular topics were: NOD mouse, natural killer (NK) T cell and interleukin-4. The latest trends showed that in the first cluster, the newest keyword was “double-negative T (DNT) cell” which appeared in 2011 37 times. In the second cluster, the new focus of “clinical research” was T2DM, which appeared in 2014 94 times, T-cell immunoglobulin and mucin domain (TIM), which appeared in 2014 26 times, and obesity, which appeared in 2014 27 times. As for cluster 3, the focus of “treatment” was islet transplantation, which appeared in 2009 27 times (S2 Table).

## Discussion

### Trend in DM and T cells research

The United States and Canada made the highest contributions to the global publications in terms of total number of publications as well as citation frequency and H-index. The Unites States and Canada started relatively early with studying DM and T cells. As shown in Fig 2B, from 1997, which is the first year that DM and T cells co-occurred in the database, publications from the United States and Canada accounted for a considerable proportion (40.74% and 22.22%, respectively). The conditions for basic experiments in the immunology field and clinical trials are better in developed countries, which include advanced equipment and techniques, sufficient funding and better scientific research systems. Therefore, scholars from the United States and Canada have a leading position in the field of DM and T cells research.

China was 5th when ranked for the total number of publications, but 11th for both citation frequency and H-index. The total number of citations and H-index of a country represent its academic impact and quality of publications. This discrepancy between the academic impact and quantity of publications may be due to the following reasons. First, studies on DM and T cells started late (in 2004) in China with the inflection point in 2008. Discoveries are difficult when following the steps of others. Besides, it takes years for the citation frequency of a publication to increase to a considerable number, even if the reported findings are important. Second, the academic evaluation system in China puts an excessive pressure on the quantity of publications[16], which forces Chinese researchers and doctors to publish articles quickly, thereby sacrificing the quality of studies. Improvements in this policy are necessary to keep up with global publications in this field.

The logistic model fitting curves showed that the inflection point of China (2008) was late because of the late start of research in 2004, compared to 1997 for the United States and Canada. When the cumulative number of publications reaches the inflection point, the rate of the number of publications changes from positive to negative. The global inflection point was in 2000, therefore the rate of increase in literature in this field has gradually slowed down since then. There are fewer studies published every year and new discoveries in this field are more difficult than before.

The Journal of Immunology, Diabetes, Journal of Autoimmunity, Proceedings of National Academy of Sciences USA, and Clinical Experimental Immunology published the most studies on DM and T cells. Future discoveries in this field are likely to be reported by the aforementioned journals.

Institutes in the United States were the leading organizations in DM and T cells research, which was consistent with the leadership of the United States in global publications. More than half (8) of the top 15 institutes were located in the United States. Meanwhile, Canadian and European institutes were also influential with 2 and 5 institutes in the top 15 list, respectively. This suggests that creating first-class research institutes is fundamental to improve the academic level of a country.

Santamaria P., Roep B.O. and Peakman M. were the top 3 authors who published the most studies in the field of DM and T cells. Santamaria P. focused on the pathogenesis of autoimmune DM, especially on the role of regulatory T cells[17] and CD8(+) T cells[18] in the autoimmune process, while Roep B.O. and Peakman M. were both making an impact by studying the homeostasis of auto-reactive T cells and regulatory T cells in T1DM[19,20]. These authors were pioneers in DM and T cells research. Their research is likely to have a tremendous impact on future developments in this field. Their new publications should be closely monitored to obtain the latest advancement on DM and T cells research.

### Research focused on DM and T cells

The highest citation frequency is related with the biggest academic impact of the publications. Reviewing those frequently citied publications provides us partly with their research focus. In the cluster of “pathogenesis”, the publication entitled “Following a diabetogenic T cell from genesis through pathogenesis” was cited 124 times. It was published in *Cell* in 1993. The authors generated a transgenic mouse strain with the rearranged T cell receptor gene from the diabetogenic T cell clone BDC2.5 derived from a NOD mouse. Insulitis and DM were induced in this mouse strain and these mice were not protected by MHC E molecules like the NOD mice[21]. In the cluster of “clinical research”, the publication entitled “B7/CD28 co-stimulation is essential for the homeostasis of the CD4+CD25+ immunoregulatory T cells that control autoimmune diabetes” was cited 117 times. It was published in Immunity in 2000. This publication reported that both B7-1/B7-2-deficient and CD28-deficient NOD mice had a profound decrease in the immunoregulatory CD4+CD25+ T cells and severe autoimmune DM, which could be prevented or delayed by transferring these regulatory T cells[22]. In the cluster of “treatment”, the publication entitled “Autoreactive T cell responses show proinflammatory polarization in diabetes but a regulatory phenotype in health” was cited 68 times. It was published in the Journal of Clinical Investigation in 2004. The researchers found that the quality of autoimmune T cells in patients with T1DM was affected and showed significant polarization toward a proinflammatory Th1 phenotype. Nondiabetic, HLA-matched control subjects also responded against islet peptides, but showed extreme T regulatory cell bias. Therefore, they concluded that the development of T1DM depends on the imbalanced auto-reactive Th1 and Treg cells, which may be manipulated by immune intervention[20]. Those studies changed the traditional concepts and brought the understanding of the pathogenesis of DM to a new level. These findings are milestones in DM and T cells research and cornerstones for future studies.

According to the bibliometric analysis, T2DM, TIM and obesity may be the next popular topics in this field. The autoimmune nature of T1DM has been widely accepted, therefore the relation between T cells and T1DM has been the main focus in this research field. However, recent research has shown that T cells also play an important role in various steps in the development of T2DM. Dysregulation of CD4+CXCR5+ T cells and production of excessive proinflammatory cytokines may play an important part in the mechanism of chronic low-grade inflammation of insulin resistance[4,23]. The increased risk of T2DM due to TCR repertoire variation also implicates a role for autoimmunity[24]. Moreover, the effects of T cells can also be found in the development of various DM-related complications such as atherosclerosis[5], nephropathy[6], retinopathy[25] and neuropathy[7]. Recent studies have shown that *in vivo* blockage of TIM-3 using monoclonal antibodies could enhance the onset frequency of autoimmune DM in an adoptive transfer model of disease[26]. Expression of TIM-4mRNA was found to significantly increase the number of mononuclear cells in T2DM patients[27]. Those studies implicated that TIM is likely involved in the pathogenesis of DM and might be a potential therapeutic target. Besides, activities of T cells may be the underlying mechanism of the relevance between obesity and DM[28]. Scientists in the field of T2DM and T cells, TIM and obesity may achieve some important research findings and publish high-level publications in the future.

### Strengths and limitations

Publications on DM and T cells research in this study were retrieved from the Web of Science database. The bibliometric analysis was relatively objective and comprehensive. However, there were still some limitations in this study. Publications in non-English languages were not included in the database and analyzed. Therefore, important non-English research studies on DM and T cells may have been excluded. Additionally, research published in 2017 was not included in this study, so the analysis of popular topics did not include the keywords of 2017. Future research should address the latest published studies and other non-English studies.

## Conclusion

This study presented the global trend in DM and T cells research. The United States had the leading position in global research in this field by contributing the most. There was a discrepancy between the academic impact and the quantity of publications from China. Publications on DM and T cells research will decrease and it will be more difficult to make discoveries than before. The newest studies could be found in the Journal of Immunology. Santamaria P., Roep B.O., and Peakman M. are good candidates for potential research collaborations in this field. T2DM, TIM and obesity are the latest popular topics and scholars currently focusing on these topics may be pioneers in this field and will be initiators for future studies.

## Supporting information

S1 Table Details of references by cluster in VOSviewer. (PDF)

S2 Table Details of group items by cluster in VOSviewer. (PDF)
