# Supplementary material for: Publication trends of research on diabetes mellitus and T cells (1997–2016): A 20-year bibliometric study
Source: PLoS One. 2017 Sep 19;12(9):e0184869. doi: 10.1371/journal.pone.0184869 (PMC5604989; doi:10.1371/journal.pone.0184869)
Supplement: S1 Table — (PDF) [file pone.0184869.s001.pdf]

Supplemental Table 1 Details of References by Cluster in VOSviewer

id	label	weight <Citations>	weight<Co-citations>	cluster
111	"achaorbea h, 1987, p natlacadsciusa, v84, p2435, doi 10.1073/pnas.84.8.2435"	25	431	1
395	"alleva dg, 2001, j clin invest, v107, p173, doi 10.1172/jci8525"	32	454	3
514	"amrani a, 2000, nature, v406, p739, doi 10.1038/35021081"	56	908	3
546	"anderson b, 1999, p natlacadsciusa, v96, p9311, doi 10.1073/pnas.96.16.9311"	37	504	1
566	"andersonms, 2005, annu rev immunol, v23, p447, doi 10.1146/annurev.immunol.23.021704.115643"	96	1176	2
592	"andrei, 1996, p natlacadsciusa, v93, p2260, doi 10.1073/pnas.93.6.2260"	39	509	2
727	"arif s, 2004, j clin invest, v113, p451, doi 10.1172/jci200419585"	68	814	3
801	"asano m, 1996, j exp med, v184, p387, doi 10.1084/jem.184.2.387"	33	549	2
884	"atkinson ma, 1992, lancet, v339, p458, doi 10.1016/0140-6736(92)91061-c"	44	496	3
889	"atkinson ma, 1994, j clin invest, v94, p2125, doi 10.1172/jci117567"	36	466	3
890	"atkinson ma, 1994, new engl j med, v331, p1428"	32	269	3
892	"atkinson ma, 1999, nat med, v5, p601, doi 10.1038/9442"	28	345	2
893	"atkinson ma, 2001, lancet, v358, p221, doi 10.1016/s0140-6736(01)05415-0"	36	306	2
1000	"bachjf, 1994, endocr rev, v15, p516, doi 10.1210/er.15.4.516"	55	720	1
1007	"bachjf, 2001, annu rev immunol, v19, p131, doi 10.1146/annurev.immunol.19.1.131"	24	351	2
1072	"baecher-allan c, 2001, j immunol, v167, p1245"	23	236	2
1097	"baekkeskov s, 1990, nature, v347, p151, doi 10.1038/347151a0"	40	606	3

1163	"balasa b, 1997, j immunol, v159, p4620"	22	301	1
1406	"baxter ag, 1997, diabetes, v46, p572, doi 10.2337/diabetes.46.4.572"	22	278	1
1486	"belghith m, 2003, nat med, v9, p1202, doi 10.1038/nm924"	40	662	2
1537	"bendelac a, 1987, j exp med, v166, p823, doi 10.1084/jem.166.4.823"	75	1184	1
1574	"bennett cl, 2001, nat genet, v27, p20"	23	390	2
1639	"bergman b, 1994, diabetes, v43, p197, doi 10.2337/diabetes.43.2.197"	26	320	1
1949	"bluestone ja, 2010, nature, v464, p1293, doi 10.1038/nature08933"	28	210	2
2031	"boitard c, 1989, j exp med, v169, p1669, doi 10.1084/jem.169.5.1669"	37	704	1
2179	"bottazzo gf, 1985, new engl j med, v313, p353, doi 10.1056/nejm198508083130604"	36	495	3
2473	"brusko t, 2007, diabetes, v56, p604, doi 10.2337/db06-1248"	42	493	2
2476	"brusko tm, 2005, diabetes, v54, p1407, doi 10.2337/diabetes.54.5.1407"	72	886	2
2730	"cameronmj, 1997, j immunol, v159, p4686"	24	297	1
2774	"candeias s, 1991, p natlacadsciusa, v88, p6167, doi 10.1073/pnas.88.14.6167"	20	269	1
2877	"carrascomarin e, 1996, j immunol, v156, p450"	20	230	1
2945	"castano l, 1990, annu rev immunol, v8, p647, doi 10.1146/annurev.immunol.8.1.647"	56	602	1
3193	"chatenoud l, 1994, p natlacadsciusa, v91, p123, doi 10.1073/pnas.91.1.123"	28	462	2
3195	"chatenoud l, 1997, j immunol, v158, p2947"	20	359	2
3197	"chatenoud l, 2001, immunol rev, v182, p149, doi 10.1034/j.1600-065x.2001.1820112.x"	21	324	2
3322	"chenwj, 2003, j exp med, v198, p1875, doi 10.1084/jem.20030152"	35	577	2
3366	"chenzb, 2005, j exp med, v202, p1387, doi 10.1084/jem.20051409"	39	549	2

3401	"chervonskyav, 1997, cell, v89, p17, doi 10.1016/s0092-8674(00)80178-6"	29	331	1
3549	"christiansonsw, 1993, diabetes, v42, p44, doi 10.2337/diabetes.42.1.44"	72	1105	1
3848	"coppieterskt, 2012, j exp med, v209, p51, doi 10.1084/jem.20111187"	29	241	3
3867	"corper al, 2000, science, v288, p505, doi 10.1126/science.288.5465.505"	21	241	1
4077	"d'alise am, 2008, p natlacadsciusa, v105, p19857, doi 10.1073/pnas.0810713105"	27	260	2
4175	"daniel d, 1995, eur j immunol, v25, p1056, doi 10.1002/eji.1830250430"	62	967	3
4177	"daniel d, 1996, p natlacadsciusa, v93, p956, doi 10.1073/pnas.93.2.956"	37	605	3
4181	"dankena, 2004, j immunol, v172, p5967"	28	341	3
4444	"delovitchtl, 1997, immunity, v7, p727, doi 10.1016/s1074-7613(00)80392-1"	106	1316	1
4578	"di lorenzotp, 2007, clinexpimmunol, v148, p1, doi 10.1111/j.1365-2249.2007.03328.x"	33	401	3
4635	"dilorenzotp, 1998, p natlacadsciusa, v95, p12538, doi 10.1073/pnas.95.21.12538"	41	674	1
4914	"durinovic-bello i, 2004, diabetologia, v47, p439, doi 10.1007/s00125-003-1315-1"	20	289	3
4920	"durinovicbelloi, 1996, diabetes, v45, p795, doi 10.2337/diabetes.45.6.795"	32	307	3
5043	"eisenbarthgs, 1986, new engl j med, v314, p1360"	33	249	3
5181	"endl j, 1997, j clin invest, v99, p2405, doi 10.1172/jci119423"	38	506	3
5593	"feuerer m, 2009, immunity, v31, p654, doi 10.1016/j.immuni.2009.08.023"	22	265	2
5744	"fontenotjd, 2003, natimmunol, v4, p330, doi 10.1038/ni904"	71	1107	2
5746	"fontenotjd, 2005, immunity, v22, p329, doi 10.1016/j.immuni.2005.01.016"	26	400	2
5906	"frenchmb, 1997, diabetes, v46, p34, doi 10.2337/diabetes.46.1.34"	22	327	3
6086	"gagnerault mc, 2002, j exp med, v196, p369, doi 10.1084/jem.20011353"	30	452	2

6611	"gombertjm, 1996, eur j immunol, v26, p2989, doi 10.1002/eji.1830261226"	35	486	1
6627	"gonzalez a, 1997, immunity, v7, p873, doi 10.1016/s1074-7613(00)80405-7"	29	368	1
6628	"gonzalez a, 2001, natimmunol, v2, p1117, doi 10.1038/ni738"	22	417	1
6768	"graserrt, 2000, j immunol, v164, p3913"	34	592	1
6810	"green ea, 2002, immunity, v16, p183, doi 10.1016/s1074-7613(02)00279-0"	20	266	2
6811	"green ea, 2003, p natlacadsciusa, v100, p10878, doi 10.1073/pnas.1834400100"	33	521	2
6841	"gregori s, 2003, j immunol, v171, p4040"	55	930	2
6904	"grinberg-bleyer y, 2010, j exp med, v207, p1871, doi 10.1084/jem.20100209"	22	328	2
6963	"groux h, 1997, nature, v389, p737"	32	454	2
7271	"hammondkjl, 1998, j exp med, v187, p1047, doi 10.1084/jem.187.7.1047"	30	328	1
7282	"han by, 2005, nat med, v11, p645, doi 10.1038/nm1250"	22	356	3
7468	"harrisonlc, 1993, lancet, v341, p1365, doi 10.1016/0140-6736(93)90940-i"	23	320	3
7471	"harrisonlc, 1996, j exp med, v184, p2167, doi 10.1084/jem.184.6.2167"	20	275	3
7521	"haskins k, 1988, diabetes, v37, p1444, doi 10.2337/diabetes.37.10.1444"	35	387	1
7523	"haskins k, 1989, p natlacadsciusa, v86, p8000, doi 10.1073/pnas.86.20.8000"	45	571	1
7524	"haskins k, 1990, science, v249, p1433, doi 10.1126/science.2205920"	71	1008	1
7525	"haskins k, 1996, diabetes, v45, p1299, doi 10.2337/diabetes.45.10.1299"	28	372	3
7527	"haskins k, 2005, advimmunol, v87, p123, doi 10.1016/s0065-2776(05)87004-x"	21	135	3
7552	"hattori m, 1986, science, v231, p733, doi 10.1126/science.3003909"	21	286	1
7582	"hawkescj, 2000, diabetes, v49, p356, doi 10.2337/diabetes.49.3.356"	23	240	3
7757	"herbelin a, 1998, j immunol, v161, p2620"	26	448	2

7768	"herman ae, 2004, j exp med, v199, p1479, doi 10.1084/jem.20040179"	38	635	2
7801	"herold kc, 2002, new engl j med, v346, p1692, doi 10.1056/nejmoa012864"	46	730	2
7804	"herold kc, 2005, diabetes, v54, p1763, doi 10.2337/diabetes.54.6.1763"	29	473	2
8008	"hoglund p, 1999, j exp med, v189, p331, doi 10.1084/jem.189.2.331"	75	996	1
8106	"hong s, 2001, nat med, v7, p1052, doi 10.1038/nm0901-1052"	29	407	1
8135	"hori s, 2003, science, v299, p1057, doi 10.1126/science.1079490"	70	1085	2
8158	"horwitzms, 1998, nat med, v4, p781, doi 10.1038/nm0798-781"	30	302	3
8361	"hultgren b, 1996, diabetes, v45, p812, doi 10.2337/diabetes.45.6.812"	24	279	1
8672	"itoh n, 1993, j clin invest, v92, p2313, doi 10.1172/jci116835"	31	464	3
8759	"jaeckel e, 2005, diabetes, v54, p306"	22	361	2
9108	"judkowski v, 2001, j immunol, v166, p908"	38	461	1
9185	"kagi d, 1997, j exp med, v186, p989, doi 10.1084/jem.186.7.989"	27	452	1
9269	"kanagawa o, 1998, p natlacadsciusa, v95, p1721, doi 10.1073/pnas.95.4.1721"	22	306	1
9490	"katz j, 1993, eur j immunol, v23, p3358, doi 10.1002/eji.1830231244"	45	734	1
9492	"katzjd, 1993, cell, v74, p1089, doi 10.1016/0092-8674(93)90730-e"	124	1548	1
9494	"katzjd, 1995, science, v268, p1185, doi 10.1126/science.7761837"	68	911	1
9505	"kaufman dl, 1993, nature, v366, p69, doi 10.1038/366069a0"	98	1369	1
9562	"kay twh, 1996, j immunol, v157, p3688"	20	386	1
9639	"kentsc, 2005, nature, v435, p224, doi 10.1038/nature03625"	40	464	3
9673	"keymeulen b, 2005, new engl j med, v352, p2598, doi 10.1056/nejmoa043980"	33	515	2
9710	"khattri r, 2003, natimmunol, v4, p337, doi 10.1038/ni909"	42	802	2

9741	"kikutani h, 1992, advimmunol, v51, p285, doi 10.1016/s0065-2776(08)60490-3"	43	487	1
9834	"king c, 2004, cell, v117, p265, doi 10.1016/s0092-8674(04)00335-6"	26	290	2
9885	"kishimoto h, 2001, natimmunol, v2, p1025, doi 10.1038/ni726"	38	494	1
10354	"krishnamurthy b, 2006, j clin invest, v116, p3258, doi 10.1172/jci29602"	27	390	3
10470	"kukreja a, 2002, j clin invest, v109, p131, doi 10.1172/jci13605"	84	1110	2
10540	"kurrermo, 1997, p natlacadsciusa, v94, p213, doi 10.1073/pnas.94.1.213"	36	545	1
10826	"latekrr, 2000, immunity, v12, p699, doi 10.1016/s1074-7613(00)80220-4"	23	240	1
10856	"lawsonjm, 2008, clinexpimmunol, v154, p353, doi 10.1111/j.1365-2249.2008.03810.x"	27	352	2
11058	"lehuen a, 1998, j exp med, v188, p1831, doi 10.1084/jem.188.10.1831"	32	414	1
11132	"lenschowdj, 1996, immunity, v5, p285, doi 10.1016/s1074-7613(00)80323-4"	21	273	1
11151	"lepault f, 2000, j immunol, v164, p240"	36	528	2
11395	"liblaurs, 1995, immunol today, v16, p34, doi 10.1016/0167-5699(95)80068-9"	31	361	1
11397	"liblaurs, 2002, immunity, v17, p1, doi 10.1016/s1074-7613(02)00338-2"	23	284	3
11417	"liebermansm, 2003, p natlacadsciusa, v100, p8384, doi 10.1073/pnas.0932778100"	62	882	3
11419	"liebermansm, 2003, tissue antigens, v62, p359, doi 10.1034/j.1399-0039.2003.00152.x"	21	342	3
11420	"liebermansm, 2004, j immunol, v173, p6727"	28	512	3
11524	"lindley s, 2005, diabetes, v54, p92, doi 10.2337/diabetes.54.1.92"	88	1041	2
11650	"liuwh, 2006, j exp med, v203, p1701, doi 10.1084/jem.20060772"	46	609	2

11743	"lohmann t, 1994, lancet, v343, p1607, doi 10.1016/s0140-6736(94)93061-9"	22	267	3
11786	"long sa, 2010, diabetes, v59, p407, doi 10.2337/db09-0694"	36	496	2
11967	"lund t, 1990, nature, v345, p727, doi 10.1038/345727a0"	21	309	1
12243	"makino s, 1980, experimental animals (tokyo), v29, p1"	33	454	1
12300	"mallone r, 2007, diabetes, v56, p613, doi 10.2337/db06-1419"	39	540	3
12625	"marwahaak, 2010, j immunol, v185, p3814, doi 10.4049/jimmunol.1001860"	21	249	2
12710	"mathis d, 2001, nature, v414, p792, doi 10.1038/414792a"	22	212	1
12876	"mcclymontsa, 2011, j immunol, v186, p3918, doi 10.4049/jimmunol.1003099"	22	292	2
13069	"mellanbyrj, 2007, immunology, v121, p15, doi 10.1111/j.1365-2567.2007.02546.x"	24	348	2
13270	"miller bj, 1988, j immunol, v140, p52"	51	857	1
13393	"miyazaki t, 1990, nature, v345, p722, doi 10.1038/345722a0"	20	321	1
13531	"monti p, 2007, j immunol, v179, p5785"	28	353	3
13695	"moriyama h, 2003, p natlacadsciusa, v100, p10376, doi 10.1073/pnas.1834450100"	22	397	3
13809	"mueller r, 1996, j exp med, v184, p1093, doi 10.1084/jem.184.3.1093"	27	441	1
13816	"muir a, 1995, j clin invest, v95, p628, doi 10.1172/jci117707"	24	358	3
13978	"nagata m, 1994, j immunol, v152, p2042"	38	761	1
14026	"nakamura k, 2001, j exp med, v194, p629, doi 10.1084/jem.194.5.629"	25	434	2
14041	"nakano n, 1991, j exp med, v173, p1091, doi 10.1084/jem.173.5.1091"	21	371	1
14047	"nakayama m, 2005, nature, v435, p220, doi 10.1038/nature03523"	62	784	3
14116	"naumovyn, 2001, p natlacadsciusa, v98, p13838, doi 10.1073/pnas.251531798"	24	362	1
14444	"noorchashm h, 1997, diabetes, v46, p941, doi 10.2337/diabetes.46.6.941"	26	240	1

14609	"ohaships, 1991, cell, v65, p305, doi 10.1016/0092-8674(91)90164-t"	29	399	1
14714	"oldstonemba, 1991, cell, v65, p319, doi 10.1016/0092-8674(91)90165-u"	20	233	1
14983	"palmer jp, 1983, science, v222, p1337, doi 10.1126/science.6362005"	28	374	3
15003	"panagiotopoulos c, 2003, diabetes, v52, p2647, doi 10.2337/diabetes.52.11.2647"	21	364	3
15027	"paninabordignon p, 1995, j exp med, v181, p1923, doi 10.1084/jem.181.5.1923"	42	680	3
15222	"peakman m, 1999, j clin invest, v104, p1449, doi 10.1172/jci7936"	21	257	3
15313	"pengyf, 2004, p natlacadsciusa, v101, p4572, doi 10.1073/pnas.0400810101"	22	306	2
15373	"pescovitz md, 2009, new engl j med, v361, p2143, doi 10.1056/nejmoa0904452"	22	157	2
15414	"petersonjd, 1996, diabetes, v45, p328, doi 10.2337/diabetes.45.3.328"	31	503	1
15575	"pinkseggm, 2005, p natlacadsciusa, v102, p18425, doi 10.1073/pnas.0508621102"	37	516	3
15701	"pop sm, 2005, j exp med, v201, p1333, doi 10.1084/jem.20042398"	47	659	2
15917	"putnam al, 2005, j autoimmun, v24, p55, doi 10.1016/j.jaut.2004.11.004"	41	529	2
15918	"putnam al, 2009, diabetes, v58, p652, doi 10.2337/db08-1168"	21	264	2
15999	"rabinovitch a, 1994, diabetes, v43, p613, doi 10.2337/diabetes.43.5.613"	31	414	1
16142	"rapoportmj, 1993, j exp med, v178, p87, doi 10.1084/jem.178.1.87"	45	613	1
16192	"razi, 2001, lancet, v358, p1749, doi 10.1016/s0140-6736(01)06801-5"	20	335	3
16202	"read s, 2000, j exp med, v192, p295, doi 10.1084/jem.192.2.295"	25	376	2
16272	"reich ep, 1989, nature, v341, p326, doi 10.1038/341326a0"	20	363	1
16291	"reijonen h, 2002, diabetes, v51, p1375, doi 10.2337/diabetes.51.5.1375"	37	477	3

16604	"roepbo, 1996, diabetes, v45, p1147, doi 10.2337/diabetes.45.9.1147"	36	409	3
16608	"roepbo, 1999, j autoimmun, v13, p267, doi 10.1006/jaut.1999.0312"	27	260	3
16613	"roepbo, 2003, diabetologia, v46, p305, doi 10.1007/s00125-003-1089-5"	35	369	3
16896	"rudy g, 1995, mol med, v1, p625"	25	324	3
17066	"sakaguchi s, 1995, j immunol, v155, p1151"	54	705	2
17073	"sakaguchi s, 2004, annu rev immunol, v22, p531, doi 10.1146/annurev.immunol.21.120601.141122"	33	447	2
17075	"sakaguchi s, 2005, natimmunol, v6, p345, doi 10.1038/ni1178"	31	364	2
17079	"sakaguchi s, 2008, cell, v133, p775, doi 10.1016/j.cell.2008.05.009"	21	189	2
17145	"salomon b, 2000, immunity, v12, p431, doi 10.1016/s1074-7613(00)80195-8"	117	1603	2
17230	"santamaria p, 1995, j immunol, v154, p2494"	28	512	1
17456	"schlootnc, 1997, diabetologia, v40, p564, doi 10.1007/s001250050716"	25	335	3
17462	"schlootnc, 2003, j autoimmun, v21, p365, doi 10.1016/s0896-8411(03)00111-2"	20	211	3
17483	"schmidt d, 1997, j exp med, v186, p1059, doi 10.1084/jem.186.7.1059"	30	526	1
17507	"schneider a, 2008, j immunol, v181, p7350"	37	461	2
17789	"serreze dv, 1993, j immunol, v150, p2534"	32	337	1
17792	"serreze dv, 1994, diabetes, v43, p505, doi 10.2337/diabetes.43.3.505"	68	1077	1
17796	"serreze dv, 1996, j exp med, v184, p2049, doi 10.1084/jem.184.5.2049"	48	558	1
17801	"serreze dv, 1998, j immunol, v161, p3912"	30	294	1
17806	"serreze dv, 2001, curr direct autoimmu, v4, p31"	22	272	1
17824	"setoguchi r, 2005, j exp med, v201, p723, doi 10.1084/jem.20041982"	22	420	2

17837	"seyfert-margolis v, 2006, diabetes, v55, p2588, doi 10.2337/db05-1378"	23	228	3
17893	"sharif s, 2001, nat med, v7, p1057, doi 10.1038/nm0901-1057"	34	480	1
17973	"shevachem, 2000, annu rev immunol, v18, p423, doi 10.1146/annurev.immunol.18.1.423"	20	266	2
17977	"shevachem, 2002, nat rev immunol, v2, p389, doi 10.1038/nri821"	34	490	2
17988	"shifd, 2001, p natlacadsciusa, v98, p6777, doi 10.1073/pnas.121169698"	20	276	1
18031	"shimizu j, 1993, j immunol, v151, p1723"	23	431	1
18075	"shizuru ja, 1988, science, v240, p659, doi 10.1126/science.2966437"	25	363	1
18123	"sibleyrk, 1985, lab invest, v53, p132"	29	444	3
18301	"skowera a, 2008, j clin invest, v118, p3390, doi 10.1172/jci35449"	37	401	3
18489	"somoza n, 1994, j immunol, v153, p1360"	23	390	3
18610	"stadinskibd, 2010, natimmunol, v11, p225, doi 10.1038/ni.1844"	38	367	3
18626	"standifer ne, 2006, diabetes, v55, p3061, doi 10.2337/db06-0066"	21	351	3
19016	"suri-payer e, 1998, j immunol, v160, p1212"	20	340	2
19100	"szanya v, 2002, j immunol, v169, p2461"	38	549	2
19188	"takahashi t, 1998, intimmunol, v10, p1969, doi 10.1093/intimm/10.12.1969"	24	385	2
19192	"takaki t, 2006, j immunol, v176, p3257"	20	332	3
19298	"tang q, 2008, immunity, v28, p687, doi 10.1016/j.immuni.2008.03.016"	60	764	2
19301	"tang qz, 2003, j immunol, v171, p3348"	22	387	2
19303	"tang qz, 2004, j exp med, v199, p1455, doi 10.1084/jem.20040139"	59	783	2
19306	"tang qz, 2006, natimmunol, v7, p83, doi 10.1038/ni1289"	27	410	2

19349	"tarbellkv, 2004, j exp med, v199, p1467, doi 10.1084/jem.20040180"	36	579	2
19350	"tarbellkv, 2007, j exp med, v204, p191, doi 10.1084/jem.20061631"	25	337	2
19470	"thebault-baumont k, 2003, j clin invest, v111, p851, doi 10.1172/jci200316584"	25	444	3
19562	"thornton am, 1998, j exp med, v188, p287, doi 10.1084/jem.188.2.287"	39	716	2
19603	"tian jd, 1996, j exp med, v183, p1561, doi 10.1084/jem.183.4.1561"	38	603	3
19604	"tian jd, 1996, nat med, v2, p1348, doi 10.1038/nm1296-1348"	20	312	2
19639	"tisch r, 1993, nature, v366, p72, doi 10.1038/366072a0"	89	1295	1
19642	"tisch r, 1996, cell, v85, p291, doi 10.1016/s0092-8674(00)81106-x"	96	1253	1
19655	"tivolea, 1995, immunity, v3, p541, doi 10.1016/1074-7613(95)90125-6"	21	290	2
19664	"todd ja, 1987, nature, v329, p599, doi 10.1038/329599a0"	37	440	1
19675	"todd ja, 2001, immunity, v15, p387, doi 10.1016/s1074-7613(01)00202-3"	27	381	1
19676	"todd ja, 2007, nat genet, v39, p857, doi 10.1038/ng2068"	21	193	2
19698	"toma a, 2005, p natlacadsciusa, v102, p10581, doi 10.1073/pnas.0504230102"	25	437	3
19817	"trembleau s, 1995, j exp med, v181, p817, doi 10.1084/jem.181.2.817"	21	279	1
19849	"tritt m, 2008, diabetes, v57, p113, doi 10.2337/db06-1700"	28	417	2
19860	"trudeaujd, 2003, j clin invest, v111, p217, doi 10.1172/jci200316409"	51	752	3
19976	"turley s, 2003, j exp med, v198, p1527, doi 10.1084/jem.20030966"	24	401	2
20026	"ueda h, 2003, nature, v423, p506, doi 10.1038/nature01621"	34	391	2
20421	"velthuisjh, 2010, diabetes, v59, p1721, doi 10.2337/db09-1486"	23	276	3
20444	"verdaguer j, 1996, j immunol, v157, p4726"	30	525	1

20445	"verdaguer j, 1997, j exp med, v186, p1663, doi 10.1084/jem.186.10.1663"	70	998	1
20456	"verge cf, 1996, diabetes, v45, p926, doi 10.2337/diabetes.45.7.926"	24	306	3
20501	"viglietta v, 2002, j clin invest, v109, p895, doi 10.1172/jci14114"	27	321	3
20502	"viglietta v, 2004, j exp med, v199, p971, doi 10.1084/jem.20031579"	25	324	2
20693	"vysetj, 1996, cell, v85, p311, doi 10.1016/s0092-8674(00)81110-1"	20	246	1
20840	"wang b, 1996, eur j immunol, v26, p1762, doi 10.1002/eji.1830260815"	42	784	1
20844	"wang b, 2001, j exp med, v194, p313, doi 10.1084/jem.194.3.313"	22	258	1
21077	"wegmanndr, 1994, eur j immunol, v24, p1853, doi 10.1002/eji.1830240820"	35	513	3
21262	"wicker ls, 1986, diabetes, v35, p855, doi 10.2337/diabetes.35.8.855"	38	523	1
21267	"wicker ls, 1994, diabetes, v43, p500, doi 10.2337/diabetes.43.3.500"	64	1021	1
21269	"wicker ls, 1995, annu rev immunol, v13, p179, doi 10.1146/annurev.immunol.13.1.179"	53	599	1
21270	"wicker ls, 1996, j clin invest, v98, p2597, doi 10.1172/jci119079"	24	356	3
21317	"willcox a, 2009, clinexpimmunol, v155, p173, doi 10.1111/j.1365-2249.2008.03860.x"	34	394	3
21381	"wilsonsb, 1998, nature, v391, p177"	35	350	1
21449	"wogensen l, 1994, j exp med, v179, p1379, doi 10.1084/jem.179.4.1379"	24	350	1
21499	"wong fs, 1996, j exp med, v183, p67, doi 10.1084/jem.183.1.67"	69	1139	1
21505	"wong fs, 1999, nat med, v5, p1026, doi 10.1038/12465"	62	872	3
21579	"wuaj, 2002, p natlacadsciusa, v99, p12287, doi 10.1073/pnas.172382999"	42	593	2
21763	"yagi h, 1992, eur j immunol, v22, p2387, doi 10.1002/eji.1830220931"	24	416	1

21813	"yamanouchi j, 2007, nat genet, v39, p329, doi 10.1038/ng1958"	34	445	2
22116	"yoonjw, 1999, science, v284, p1183, doi 10.1126/science.284.5417.1183"	23	305	3
22160	"you s, 2005, diabetes, v54, p1415, doi 10.2337/diabetes.54.5.1415"	45	761	2
22208	"yulp, 2000, p natlacadsciusa, v97, p1701, doi 10.1073/pnas.040556697"	23	317	3
22351	"zekzer d, 1998, j clin invest, v101, p68, doi 10.1172/jci119878"	34	569	3
22527	"zhangzj, 1991, p natlacadsciusa, v88, p10252, doi 10.1073/pnas.88.22.10252"	24	399	3
22657	"zhouxy, 2009, natimmunol, v10, p1000, doi 10.1038/ni.1774"	24	319	2
22714	"zipris d, 1991, j immunol, v146, p3763"	25	188	1
22756	"zucchelli s, 2005, immunity, v22, p385, doi 10.1016/j.immuni.2005.01.015"	22	270	1
