# Supplementary material for: Publication trends of research on diabetes mellitus and T cells (1997–2016): A 20-year bibliometric study
Source: PLoS One. 2017 Sep 19;12(9):e0184869. doi: 10.1371/journal.pone.0184869 (PMC5604989; doi:10.1371/journal.pone.0184869)
Supplement: S2 Table — (PDF) [file pone.0184869.s002.pdf]

Supplemental Table 2. Details of Group Items by Cluster in VOSviewer

id	label	weight<Occurrences>	weight<Co-occurrences>	cluster	score<Avg. pub. year>
1	accumulation	41	847	1	2008.293
2	administration	93	2211	1	2006.333
3	adoptive transfer	84	1967	1	2008.048
4	adult	44	1170	2	2010.614
5	aim	53	1472	2	2010.283
6	alpha	123	2855	1	2006.26
7	alpha galcer	31	612	1	2005.871
8	alteration	54	1181	2	2010.093
9	animal	77	1669	1	2006.325
10	antigen	344	8448	3	2006.363
11	apc	58	1517	1	2007.517
12	assay	125	3397	3	2008.336
13	association	96	2153	2	2008.167
14	autoantibody	131	3384	3	2007.458
15	autoantigen	213	5271	3	2005.061
16	autoimmune diabete	304	6306	1	2006.023
17	b cell	129	2886	1	2006.938
18	b lymphocyte	26	670	1	2003.462
19	bdc	50	1032	1	2009.32
20	beta cell antigen	36	737	3	2004.639
21	beta cell function	35	821	2	2008.771
22	binding	46	1150	3	2006
23	c57bl	56	1369	1	2006.304
24	case	49	1255	2	2009.327
25	cd127	27	823	2	2012.037
26	cd25	40	922	2	2008.175
27	cd3	62	1530	2	2008.968
28	cd45ra	29	640	2	2008.241
29	cd62l	31	649	1	2005.807
30	cd8	170	3877	1	2007.882
31	cell death	55	926	1	2006.436
32	cfa	21	603	1	2007.048
33	chain	96	2409	1	2006.104
34	change	95	2079	2	2010.084
35	child	205	5125	2	2009.259
36	chromosome	37	701	1	2005.946
37	class	75	1870	3	2006.707
38	clinical trial	36	663	2	2011.306
39	clone	79	1859	3	2003.886
40	comparison	38	966	2	2009.579
41	concentration	62	1511	2	2009.452
42	conclusion	154	4097	2	2009.825
43	control	276	6558	2	2009.044

44	control group	38	1031	2	2011.553
45	control subject	72	1930	3	2006.292
46	ctl	65	1370	1	2007
47	ctla	107	2411	2	2007.271
48	cytotoxic t lymphocyte	42	858	1	2006.191
49	cytotoxicity	35	846	3	2005
50	day	126	3529	2	2008.405
51	dcs	113	2387	1	2009.327
52	decrease	63	1440	2	2010.064
53	deficiency	86	1687	1	2007.279
54	deletion	62	1370	1	2008.484
55	dendritic cell	120	2628	1	2008.392
56	depletion	66	1509	1	2009.121
57	detection	29	699	3	2005.897
58	diabetes development	87	2003	1	2006.333
59	diabetes mellitus	158	3448	2	2010.595
60	diabetes patient	50	1212	2	2008.3
61	diabetic mouse	140	2674	1	2009.457
62	diabetic nod mouse	65	1407	1	2008.139
63	diabetic patient	196	4747	3	2006.321
64	diabetic subject	33	677	2	2007.97
65	diabetogenic	59	1286	1	2006.729
66	diabetogenic t cell	117	2404	1	2005.231
67	diagnosis	51	1265	2	2008.824
68	difference	80	2172	2	2007.638
69	dn t cell	37	778	1	2011.622
70	dose	107	2859	2	2010.794
71	dr4	41	937	3	2006.146
72	effector t cell	69	1423	2	2009.522
73	efficacy	54	1151	2	2011.019
74	epitope	217	5302	3	2007.23
75	event	66	1586	2	2007.788
76	fas	26	485	1	2005.423
77	flow cytometry	58	1451	2	2010.672
78	foxp3	163	3660	2	2009.951
79	frequency	328	7972	2	2010.25
80	fulminant type	25	519	2	2008.52
81	gad	119	3610	3	2003.361
82	gad65	56	1545	3	2004.536
83	gal	25	745	2	2010.8
84	gamma delta t cell	27	381	1	2010.444
85	glutamic acid decarboxylase	89	2588	3	2003.753
86	group	236	6300	2	2010.343
87	healthy control	83	1955	2	2009.94
88	healthy control subject	28	682	2	2007.821
89	healthy subject	30	819	2	2008.767
90	high	55	1392	2	2010.691
91	hla	98	2402	3	2007.388
92	human type	58	1335	3	2007.966

93	hyperglycemia	65	1265	1	2010.339
94	iddm	139	3649	3	1999.698
95	iddm patient	26	774	3	1999.923
96	identification	48	1130	3	2006.708
97	ifn	33	823	2	2011.303
98	igrp	79	1742	1	2010.304
99	immunization	40	1001	1	2005.1
100	importance	33	790	1	2008.242
101	incidence	68	1534	1	2007.529
102	individual	121	2584	2	2009.058
103	inhibition	86	1638	1	2008.837
104	initiation	47	1056	1	2005.404
105	injection	74	1770	1	2008.284
106	inkt cell	64	1117	1	2009.672
107	insulin dependent diabete	53	1215	3	2001.453
108	insulin dependent diabetes mellitus	40	1006	3	2000.1
109	insulin resistance	28	583	2	2010.179
110	insulitis	232	4775	1	2005.668
111	interaction	80	1472	1	2007.8
112	islet cell	37	819	1	2002.919
113	islet transplantation	27	530	3	2009.852
114	lada	32	835	2	2011.438
115	lada patient	25	548	2	2010.8
116	line	57	1647	3	2003.105
117	lymph node	70	1582	1	2006.671
118	mab	31	690	1	2004.968
119	macrophage	87	1772	1	2006.241
120	maintenance	44	951	2	2008.205
121	major histocompatibility complex	34	895	1	2005.824
122	majority	35	827	3	2006.543
123	marker	119	2653	2	2008.723
124	method	130	3314	2	2010.162
125	mhc	71	1753	1	2006.775
126	mhc class	58	1420	1	2005.448
127	mice	55	1270	1	2010.055
128	migration	60	1027	1	2007.683
129	modulation	55	1103	1	2008.109
130	molecule	213	5319	3	2005.845
131	month	65	1726	2	2009.292
132	mouse	1242	25700	1	2006.878
133	nk cell	35	767	2	2011.171
134	nk t cell	30	717	1	2001.333
135	nod	417	9418	1	2005.875
136	nod mouse	798	17064	1	2006.668
137	nod mouse model	27	512	1	2009.444
138	non obese diabetic	93	2070	1	2007.226
139	nonobese diabetic	160	3532	1	2004.219

140	nonobese diabetic mice	24	456	1	2012.083
141	nonobese diabetic mouse	107	2321	1	2003.374
142	ntreg	30	589	2	2011.433
143	null	61	1698	1	2007.918
144	obesity	27	464	2	2014
145	objective	26	782	2	2011.269
146	pancreatic lymph node	95	2253	1	2007.737
147	panel	30	744	3	2005.833
148	participant	33	1179	2	2013.424
149	pathogenic t cell	30	556	3	2006.8
150	pathway	123	2377	1	2007.504
151	patient	795	18016	2	2009.176
152	pbmc	62	1808	3	2008.274
153	peptide	503	12946	3	2006.137
154	percentage	110	2828	2	2011.164
155	perforin	21	531	1	2007.191
156	peripheral blood	106	2802	2	2008.708
157	peripheral blood mononuclear cell	61	1723	3	2007.443
158	peripheral tolerance	46	939	1	2007.261
159	phosphatase catalytic subunit related protein	21	517	1	2009.81
160	placebo	29	907	2	2011.241
161	pln	27	695	1	2009.556
162	polymorphism	86	1886	2	2009.314
163	presentation	65	1701	1	2006.154
164	prevention	75	1708	1	2006.653
165	proinsulin	62	1843	3	2006.726
166	protection	169	3945	1	2006.219
167	protective effect	38	841	1	2007.447
168	protein	184	4257	3	2005.929
169	rapamycin	21	395	2	2009.048
170	rat	172	2883	1	2005.424
171	reactivity	53	1539	3	2004.509
172	recent onset	20	539	2	2007.9
173	recent onset type	24	660	2	2009.167
174	recipient	147	3413	1	2005.993
175	recognition	65	1532	3	2006.2
176	recruitment	58	1039	1	2006.655
177	reg	33	652	2	2008.485
178	region	125	3345	3	2007.008
179	regulatory cell	27	628	1	2005.704
180	regulatory t	24	478	2	2010.75
181	regulatory t cells	36	910	2	2012.306
182	residue	40	1142	3	2005.1
183	resistance	61	1472	1	2005.049
184	response	674	16086	3	2007.068
185	risk	111	2395	2	2008.748
186	selection	80	1809	1	2005.788

187	self tolerance	40	693	2	2009.85
188	sensitivity	40	944	2	2009.3
189	sequence	57	1670	3	2004.368
190	significant difference	31	891	2	2007.226
191	specific cd4	51	1222	3	2008.353
192	specific t cell	86	2063	3	2007.535
193	specificity	92	2190	3	2006.641
194	spleen	30	777	1	2005.8
195	splenocyte	84	2001	1	2006.5
196	strain	113	2528	1	2007.115
197	streptozotocin	44	934	1	2009.182
198	stz	53	1192	1	2008.359
199	subject	174	3927	2	2008.397
200	suppression	102	2078	2	2008.99
201	suppressive function	25	670	2	2010.84
202	survival	76	1629	1	2009.474
203	susceptibility	131	3149	1	2007.076
204	t cell clone	164	3753	3	2003.933
205	t cell epitope	45	1099	3	2005
206	t cell line	59	1531	3	2002.017
207	t cell reactivity	57	1627	3	2006.193
208	t cell receptor	81	1894	1	2005.889
209	t cell response	257	6316	3	2006.751
210	t cell tolerance	29	602	1	2006.828
211	t reg	76	1493	2	2010.053
212	t reg cell	57	1138	2	2010.947
213	t regulatory cell	62	1408	2	2010.823
214	t1d development	22	409	1	2009.818
215	t1d patient	71	1644	2	2011.718
216	t1d subject	30	706	2	2010.133
217	t1dm	83	1824	2	2010.699
218	t2d	38	698	2	2014.579
219	t2dm	56	1508	2	2014.732
220	t2dm patient	29	829	2	2014.138
221	target	113	2688	3	2008.54
222	tcr	172	3662	1	2005.645
223	teff	50	1030	2	2012.58
224	tetramer	53	1250	3	2008.019
225	tgf beta	89	2197	2	2009.371
226	thymocyte	66	1407	1	2004.652
227	thymus	60	1308	1	2006.4
228	tid	29	597	1	2007.759
229	tim	26	597	2	2014.615
230	tnf	44	714	1	2006.296
231	tnf alpha	68	1523	1	2007.015
232	tolerance	214	4320	1	2008.836
233	total	21	614	2	2012.571
234	transfer	110	2432	1	2006.9
235	transgenic mouse	79	2004	1	2005.443

236	transplantation	52	1051	1	2008.654
237	treg	389	8722	2	2011.144
238	treg cell	115	2491	2	2012.017
239	treg function	23	553	2	2010.957
240	tregs	252	5886	2	2011.544
241	use	67	1536	2	2008.776
242	v alpha	56	1508	1	1999.411
243	v beta	47	992	3	2007.319
244	vivo	95	1831	1	2007.716
245	year	80	2005	2	2010.5
